# Morphological and molecular characterization of variation in common bean (*Phaseolus vulgaris* L.) germplasm from Azad Jammu and Kashmir, Pakistan

**DOI:** 10.1371/journal.pone.0265817

**Published:** 2022-04-26

**Authors:** Sidra Nasar, Kate Ostevik, Ghulam Murtaza, Mark D. Rausher

**Affiliations:** 1 Department of Botany, University of Azad Jammu and Kashmir, Muzaffarabad, Pakistan; 2 Department of Biology, Duke University, Durham, North Carolina, United States of America; 3 Department of Evolution, Ecology, and Organismal Biology, University of California Riverside, Riverside, California, United States of America; National Cheng Kung University, TAIWAN

## Abstract

*Phaseolus vulgaris*, an essential food and source of protein, is cultivated across the world. This study was carried out to investigate the diversity and population structure of 34 *P*. *vulgaris* landrace accessions collected from the Azad Jammu and Kashmir (AJ&K) regions of Pakistan. The samples were analyzed both morphologically and using genetic variation identified through RNA sequencing. Our results indicated that most genetic variation occurs among local accessions, with little genetic variation occurring between geographical regions. In addition, the accessions fell into two major genetic groups. Morphological analysis revealed that these two genetic groups differ in a number of quantitative traits, including seed length, seed width, and seed weight. One accession, DUD-11, appears to be a mixture of the two major groups genetically as well as morphologically. Among the other accessions, DUD-8, RWK-2, and NGD-1 depicted particularly high seed weight along with higher seed length, seed width, and seed yield per plant. We suggest focusing on these accessions in future breeding programs. More generally, our results provide baseline data that will be useful for crop improvement and effective cultivation practices in Pakistan.

## Introduction

The legume family, Leguminosae, is the second most important family among crop plants after Poaceae [1] and contains five *Phaseolus* species that are cultivated worldwide for the commercial production of beans. Common bean, *Phaseolus vulgaris* L. (2n = 2x = 22), is the most economically important bean species, having an 85% share in worldwide bean production, followed by *P*. *lunatus*, *P*. *coccineus*, *P*. *acutifolius* and *P*. *dumosus* [2]. In fact, common bean is the most important agricultural crop after cereals, with an annual global dry yield of more than 8741 hg/ha over 33 million ha [3]. Furthermore, *P*. *vulgaris* is considered a functional food because it is a rich source of protein, fiber, vitamins and essential minerals [4]. Because common bean is such an important crop, breeders are interested in the amount and distribution of genetic and morphological variation in the species.

Previous studies have assessed morpho-agronomic and genetic diversity in common bean and its close relative runner bean (*P*. *coccineus*). For example, Rosales-Serna et al. [5] carried out genetic analysis of Mexican common bean and runner bean landraces and cultivars by using AFLP markers. Their results showed a broad range of genetic diversity within and between Mexican bean races and that *P*. *coccineus* cultivars are clearly distinct from *P*. *vulgaris*. Likewise, Guerra-Garcia et al. [6] reported the *P*. *coccineus* domestication history and high level of genetic diversity among wild and cultivated populations of Mexico and Spain. In addition, evidence based on morphological [7], biochemical [8, 9], and molecular analyses [10, 11] suggests that there are two distinct gene pools for the common bean, known as Mesoamerican (small-seeded type) and Andean (large-seeded type). These gene pools have diverged from a wild ancestral *P*. *vulgaris* because of geographical isolation over the last approximately 100,000 years [12]. Common beans have been domesticated from each of these gene pools [13] and cultivars have been dispersed world-wide from both of these centers of origin. There are strong indications that Portuguese, Dutch, English, and/or French travelers bought the common bean with them for trading in the early 16^th^ century when they travelled through Indian subcontinent. However, there is very little known about the dissemination of common beans in the Himalayan region where a rich diversity of beans occurs [14]. Although dispersal outside of Mesoamerica and the Andes region has reduced diversity [15], most of the previous studies demonstrate substantial genetic and morphological diversity in beans, with clusters of similar cultivars that are distinct from other clusters. Many of these studies have focused on Mexican or Andean cultivars [16], while others have focused on either seed banks or cultivars in countries like India and Brazil [17–19].

*P*. *vulgaris* is grown extensively in the northern areas of Pakistan, including the Azad Jammu and Kashmir (AJ&K) regions, where the average annual yield is 0.5t/ha and can be as high as 2.5-5t/ha, under the best cultivation practices. Currently, production is unable to meet the national demand [20, 21]. Much of current production is based upon small land holdings with centuries-old customs and cultivation of local landraces [22]. The development of such landraces can lead to genetic divergence between localities, counteracting the reduction of diversity associated with dispersal bottlenecks. However, because there have been no previous investigations of genetic diversity among these land races, the amount and geographic distribution of variation available for crop improvement in Pakistan is currently unknown. There has not been even a single registered variety of common bean reported so far in Pakistan [23–25]. It is thus unknown how much genetic variation exists within the AJ&K region, whether it contains material from both centers of domestication, and particularly if that variation is sufficient for use in crop improvement [26, 27]. Thus, there is a great need to identify high yield common bean accessions that enhance the livelihood of our farmers, to make Pakistan self-sufficient for beans and proves to be profitable in foreign exchange [21].

In a broad sense, the current research aims to assess landrace genotypes that could perform better in low input agriculture systems, to conserve the selected germplasm from possible extinction, and to provide baseline data of selected germplasm for further research purposes. The specific objective of this study is to characterize morphological and genetic variation among Pakistani cultivars and regions of the country. To do this, we address the following questions: (1) How variable are important morphoagronomic traits in Pakistan? (2) What is the heritability of those traits? (3) How much genetic variation is present in Pakistani cultivars? (4) How is the morphological and genetic variation distributed geographically? (5) How does the amount of genetic variation in Pakistan compare to variation present in the *P*. *vulgaris* centers of origin in the Americas? These analyses should prove helpful in guiding breeding programs for common bean improvement in Pakistan.

## Materials and methods

Thirty-four accessions of *P*. *vulgaris* and one accession of *P*. *coccineus* were collected from AJ&K. The *P*. *vulgaris* accessions were collected from six districts with 1–4 sampling sites in each district and a single *P*. *coccineus* accession was collected from the Nagdar site in the Neelum district (see Table 1 and S1 Table for details).

**Table 1:** *Phaseolus vulgaris* accessions and geographical coordinates of sampling sites in Azad Jammu and Kashmir, Pakistan.

District	Sampling site	Latitude (N)	Longitude (E)	Accessions codes	Species
Neelum	Nagder	34˚40’47"	73˚56’14"	NGD_13	*P*. *coccineus*
				NGD_1	*P*. *vulgaris*
	Dodnyal	34˚42’08" - 34˚40’30"	74˚06’25" - 74˚05’31"	DUD_1, DUD_2, DUD_4, DUD_8, DUD_11, DUD_12	*P*. *vulgaris*
	Kel	34˚48’31" - 34˚49’27"	74˚21’06" - 74˚21’07"	KEL_2, KEL_3, KEL_5, KEL_8, KEL_11, KEL_14	*P*. *vulgaris*
	Halmat	34˚45’09"	74˚39’34"	HAL_2, HAL_6	*P*. *vulgaris*
Hattian (Leepa valley)	Gai pora	34˚18’41" - 34˚18’59"	73˚51’01"	LPA_1, LPA_3, LPA_4, LPA_5	*P*. *vulgaris*
	Nokot	34˚18’23"	73˚54’31"	LPA_6, LPA_9	*P*. *vulgaris*
Muzaffarabad	Machiyara	34˚29’58"	73˚37’02"	MAC_2, MAC_3, MAC_4	*P*. *vulgaris*
Haveli (Farwad Kahuta)	Khurshidabad	33˚55’24"	74˚10’26"	FK_1, FK_2, FK_3	*P*. *vulgaris*
	Kirni	33˚56’27"	74˚13’19"	FK_4, FK_7, FK_8, FK_9	*P*. *vulgaris*
Poonch (Rawalakot)	Chhota gala	33˚49’21"	73˚48’13"	RWK_1, RWK_2	*P*. *vulgaris*
Bagh	Saver	34˚01’31"	73˚48’57"	BG_1	*P*. *vulgaris*

Ranges for Latitude and Longitude given for sampling sites at which accessions differ in these values.

### Measurement of morphological characters

Quantitative morphological traits were measured in a field experiment involving 34 *P*. *vulgaris* accessions and the one *P*. *coccineus* accession. Seeds were planted on May 1^st^ in 2017 and 2018 in a randomized complete block design with three replicates at a location in Muzaffarabad (latitude: 34˚22’10" N, longitude: 73˚31’30" E, altitude 1,731 m). The site has an average annual rainfall of 1300 mm and a mean annual temperature of 16 ˚C. The soil in fields in Muzaffarabad is basic in nature with sufficient organic matter [28]. The field was prepared by deep ploughing, followed by row cultivation, with 80 cm between rows and 40 cm between plants within a row. Plants were allowed to twine up rope supports and weeds were removed periodically.

Thirteen quantitative morphological traits were measured on each of 10 randomly chosen plants per accession at various plant growth stages from seed germination to seed formation following methods recommended by the International Board for Plant Genetic Resources *Phaseolus L*. Descriptor list [29] (Table 2).

**Table 2:** List of investigated quantitative traits following International Board for Plant Genetic Resources list (IBPGR, 1982).

Traits	Quantitative traits measurements
Days to flowering	Days to flowering (DTF) were observed from the date of sowing to the date on which 50% of the plants in a plot had set flowers.
Plant height	After maturity, plant height (PH) was measured by using meter scale from first node up to the tip of the plant.
Leaflet length	Terminal leaflet length (LL) of third trifoliate leaf was measured in cm from Pulvinus to the leaf tip for randomly selected plants.
Leaflet width	Leaflet width (LW) of terminal third trifoliate in cm, was measured for randomly selected plants.
Stem girth	At maturity, stem girth (SG) was determined by wrapping a string around plant stem followed by measuring string using graduated meter ruler and obtaining the circumference in cm.
Pod length	Pod length (PL) in cm, was measured from the tip to the peduncle from 10 randomly selected fully expanded immature pods per plant.
Pod width	Pod width (PW) of the fully expanded pods was measured in cm by randomly selected 10 fully expanded immature pods per plant.
Seeds per pod	Total number of seeds per pod (SPP) was counted by taking average number of seeds from randomly selected pods per plant.
Pod Beak length	Pod beak length (PBL) was recorded in cm from end of last locule from randomly selected 10 pods per plant.
Seed length	Seed length (SL) was measured parallel to the hilum in mm through randomly selected ten seeds per plant by using Vernier caliper.
Seed width	Seed width (SW) was measured from hilum opposite side in mm by selecting randomly ten seeds per plant using Vernier caliper
Hundred seeds weight	Hundred dry seeds (HSW) of three randomly drawn samples from each experimental plot were taken, weighed in grams and averaged.
Seed yield per plant	Seed yield of selected plants (SYPP) from each plot was weighed and averaged to single plant basis in grams.

### RNA extraction and cDNA library construction

We use RNA-sequencing (RNAseq) of leaf tissue to determine the sequences of genes expressed in leaf tissue and identify single-nucleotide polymorphisms (SNPs) that are subsequently used to characterize patterns of genetic diversity in *P*. *vulgaris*. RNAseq is commonly used to identify SNPS for characterizing genetic diversity and constructing phylogenies [30–37]. Molecular characterization of genetic variation was conducted on one of the ten individuals from each of the 35 accessions on which morphological measurements were performed. RNA was extracted from approximately 100mg of leaf tissue from plants at the 2-leaf stage following the protocol of the Spectrum Plant Total RNA Kit (Sigma-Aldrich). After extraction, the concentration, purity and integrity of RNA were examined by using a Nanodrop apparatus (Thermo Fisher Scientific, Waltham, MA, USA) and gel electrophoresis using 1% agarose gels. cDNA libraries were prepared using the KAPA Stranded RNA-Seq library preparation kit (Roche) following the manufacturer’s protocol. A separate library was created for each accession. The quality, quantity, and size of the libraries were checked using a Qubit fluorometer (Thermo Fisher Scientific, Waltham, MA, USA) and a Bioanalyzer system (Agilent Technologies, Santa Clara, CA, USA). Libraries were sequenced (150 bp paired-end reads) over two lanes on the Illumina HiSeq 4000 platform (Illumina, Inc., San Diego, CA, USA) yielding 627 million paired reads (12–26 million per accession). The sequencing quality was examined using the FastQC tool [38]. Very low-quality bases and reads and reads containing primer or adapter contamination were removed using the software Trimmomatic 0.36 [39]. Specifically, a 4-bp sliding window was used to clip reads that fell below an average PHRED score of 5, and bases with scores below 5 were removed from the start and end of reads. Reads that were less than 25 bp after trimming were discarded. Overall, much less than 1% of reads were discarded. A reference transcriptome was constructed using data from the individual with highest coverage (LPA_9) and the trinity software [40] with the default settings. This resulted in 72,927,950 bases assembled into 74,575 transcripts.

#### Variant detection

To detect SNPs and indels among the accession, the filtered reads of each accession were aligned to the reference transcripts (75–85% of reads successfully aligned) and SNPs and indels were called using Samtools mpilup [41]. We filtered all sites (variant and invariant) for least 100 reads and a map quality score of at least 30. This yielded 12,055,420 total sites, of which 269,883 were variant (224,480 SNPs and 45,403 indels; see S1 Fig for histograms of the heterozygosity and minor allele frequencies of these variants).

We also generated a more stringently filtered set of variants. In this case, biallelic variants were filtered for a minimum minor allele frequency of 1% and complete coverage across individuals (i.e., no missing data), and thinned to a single variant per 1kb. This resulted in a total of 15,149 variants.

### Statistical analysis

Descriptive statistics were calculated for both the morphological and genetic datasets. To determine whether accessions differed for the morphological traits, we performed a standard analysis of variance (ANOVA) using Statistix 8.1 [42]. Phenotypic (V_P_) and genetic (V_G_) variance components were calculated using:

$$V_{G}=\frac{Genotype\;mean\;square\;(GMS)-Error\;mean\;square\;(EMS)}{Number\;of\;replications\;(r)}$$

$$V_{P}\;=\;V_{G}+V_{E}$$

                                Where V_E_ = Error mean square [43]

Broad-sense heritability was calculated as $H^{2}\;=\;\frac{V_{G}}{V_{P}}$ [44] with the statistical significance of the heritability assessed using an ANOVA with replicate nested within accession. The genetic relationship among accessions was examined, using the stringently filtered set of variants, in three ways. First, we performed a principal component analysis (PCA) the R package SNPRelate [45]. Second, we performed a cluster analysis, which groups accessions on the basis of similarity index, and plotted the resulting dendrogram in SNPRelate. Finally, we performed a STRUCTURE analysis [46] implemented in StrAuto [47] with 10 replicate runs with 1,000 iterations after 1,000 burn‐in iterations for each of 10 potential population clusters (K = 1–10). We used Structure Harvester [48] and the ΔK approach [49] to determine the number of population clusters that best fit our data.

To further characterize genetic diversity, we calculated nucleotide diversity (π) both within the 34 samples, as well as within and between two clusters of samples identified by STRUCTURE and PCA. Pairwise π, the proportion of nucleotides by which two sequences from different individuals differ, was calculated using an APL program written by one of the authors (MDR). This program first assigns SNP, *S*, equal to 0 if the SNP genotype was the same for the two samples, 1 if the two SNPs were homozygous for different alleles, and 0.5 otherwise. Scores were summed to produce *S*_*T*_. To correct bias caused by missing values, a corrected sum was calculated as *S*_*C*_ *= S*_*T*_ (1+ *F*), where *F* is the fraction of SNPs that were missing in at least one sample. π was then calculated as π = *S*_*C*_/*N*, where *N* is the total number of nucleotides in the transcriptome that passed filtering criteria (12,055,420). The program also calculates average π and its standard error, and performs a bootstrap analysis with 1,000 replicates to estimate confidence intervals.

To determine the extent of geographical trait differentiation, we performed a multiple analysis of variance (MANOVA) in which the factors were Among Districts (random effect), Among Sites Within Districts (nested, random effect) and Within-Sites using PROC GLM in the SAS software package, Version 9.4, for Windows. Variance components were estimated using PROC VARCOMP with the REML analysis. Variance components were converted to the proportion of variation explained by each effect. Following the MANOVA, univariate ANOVA analyses were performed.

The relationship between trait variation and genetic variation was examined by determining whether traits differed for the two major clusters identified by the analyses described above. Specifically, we performed both MANOVA and univariate ANOVA on trait values in which the main effect was cluster. We excluded the accession DUD_11 from this analysis because it fell outside the two main clusters and because our STRUCTURE analysis suggests that it is a hybrid between the genotypes represented by the two clusters.

## Results

### Morphological traits

Analysis of Variance indicates that all morphological traits differed among accessions (P < 0.001 for all traits in both years; S2 Table). Broad-sense heritability was high for all traits, ranging from 0.54 to 0.99 (Table 3).

**Table 3:** Genetic variance components and broad sense heritability for 13 morphological traits of 34 *Phaseolus vulgaris* accessions.

	Phenotypic variance		Genotypic variance		Heritability (bs)	
Traits	2017	2018	2017	2018	2017	2018
Days to flowering	4202.03	4386.06	4193.03	4383.26	0.93	0.89
Plant height	2.52	2.50	1.71	1.67	0.99	0.99
Leaflet length	2.03	2.02	1.10	1.09	0.68	0.67
Leaflet width	2.18	2.17	1.49	1.49	0.54	0.54
Stem girth	0.06	0.05	0.04	0.03	0.86	0.88
Pod length	0.04	0.04	0.04	0.03	0.69	0.69
Pod width	0.33	0.26	0.23	0.24	0.67	0.67
Pod beak length	153.85	158.25	147.82	152.41	0.87	0.85
Number seeds per pod	3.56	3.56	3.08	3.08	0.72	0.93
Seed length	1.01	1.01	0.83	0.83	0.87	0.87
Seed width	0.32	0.29	0.27	0.26	0.82	0.82
Hundred Seed weight	44.24	46.36	43.46	44.04	0.96	0.96
Seed yield per plant	18.01	21.11	16.79	18.85	0.98	0.95

Comparison of morphological traits across years revealed that for all traits there was a very strong correlation between trait value in 2017 and trait value in 2018 (S2 Fig). Correlation coefficients ranged from 0.944 to 1.0. Because of these high correlations, in subsequent analyses we used the average trait value across years to simplify the analyses (S3 Table).

There was little evidence of geographic differentiation in traits. A MANOVA analysis revealed no evidence for significant variation either among districts or among sites within districts. We performed two analyses. In the first, we included both district and site nested within district. In this model, the district effect could not be tested because there were too few error degrees of freedom. By contrast, the site effect was not significant (Wilks Lambda = 0.03326, F = 0.83, df = 65, 51.202, P = 0.761). The second model included only the district effect, which was also not significant (Wilks Lambda = 0.011, F = 1.26, df = 65, 51.202, P = 0.198).

Univariate analyses of individual traits yielded a similar conclusion. Variance components for both district and site effects were generally small and non-significant (Table 4). The only exception is for pod width, for which there was no significant site effect, and the district effect was significant when there was no site effect in the model. The combined multivariate and univariate analyses thus indicated that most variation for traits is local: it is among accessions within sites.

**Table 4:** Variance components (proportion of variance explained) for different levels of geographic variation for 13 morphological traits.

Traits	Districts	P1	P2	Site	P3	Within site
Days to flowering	0.000	0.470	0.587	0.000	0.474	1.0
Plant height	0.000	0.727	0.325	0.295	0.030	0.705
Leaflet length	0.000	0.858	0.973	0.000	0.488	1.000
Leaflet width	0.000	0.956	0.969	0.010	0.129	0.990
Stem girth	0.092	0.307	0.049	0.325	0.063	0.583
Pod length	0.000	0.492	0.781	0.000	0.960	1.000
Pod width	0.000	0.235	0.430	0.000	0.789	1.000
Pod beak length	0.434	0.020	0.0034*	0.000	0.357	0.566
Number seeds per pod	0.000	0.388	0.480	0.000	0.443	1.000
Seed length	0.081	0.116	0.218	0.000	0.822	0.919
Seed width	0.093	0.154	0.288	0.000	0.837	0.907
Hundred Seed weight	0.145	0.059	0.174	0.000	0.839	0.855
Seed yield per plant	0.000	0.609	0.552	0.003	0.572	0.997

P1: P value for test of District effect over synthesized denominator incorporation Site and Within-site effects. P2: P value for model with no Site effect included (appropriate where site effect not significant). P3: P value for Site effect in full model.

*Significant after sequential Bonferroni correction for 13 traits (i.e., at P = 0.05/13 = 0.00385).

### Patterns of genetic variation

**Fig 1:**
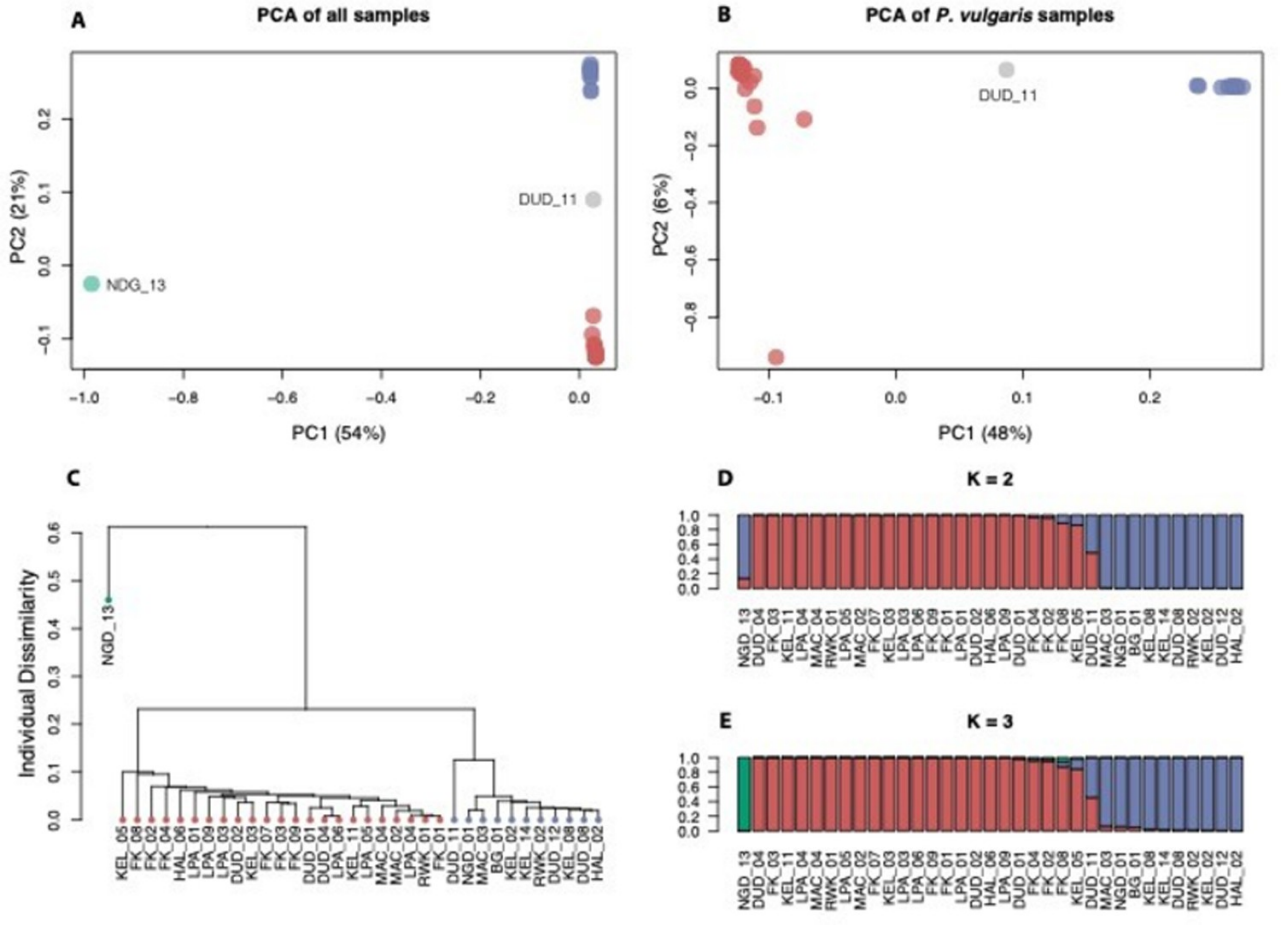
Genetic analyses of 34 *P*. *vulgaris* accessions and one *P*. *coccineus* accession (NGD_13). A. Principal component analyses on all individuals. B. Principal component analyses on the *P*. *vulgaris* individuals. C. Cluster analysis on all individuals. D. STRUCTURE plot for K = 2. E. STRUCTURE plot for K = 3. 218. Red and blue colors correspond to inferred Mesoamerican and Andean accessions, respectively.

A PCA on the *P*. *vulgaris* and *P*. *coccineus* accessions revealed that the first two principal components accounted for 75% of the genetic variation. Principal component one separates *P*. *coccineus* from *P*. *vulgaris* and principal component two separates *P*. *vulgaris* into two major groups (Fig 1A; groups are color coded). A second PCA performed on only the 34 *P*. *vulgaris* accessions also indicated that *P*. *vulgaris* is divided into two major genetic groups, this time along the first principal component, which accounts for 48% of the variation. In both analyses, accession DUD_11 falls between the two *P*. *vulgaris* groups, suggesting it may be a hybrid between the two groups.

A cluster analysis revealed groupings that are consistent with those obtained from the PCA (Fig 1C). The accessions contained within these two groups are identical with those in the two groups identified by PCA. Additionally, accession DUD_11 is an outgroup to group A and to group B, consistent with it being between the two clusters in the PCA analysis.

Finally, a STRUCTURE analysis also yielded a pattern consistent with the previous two analyses. The delta K vs. K plot (S3 Fig) shows a single peak at K = 2. Examination of the STRUCTURE plots for K = 2 and K = 3 revealed that they are very similar, except that at K = 3 the *P*. *coccineus* individual constitutes a separate group. In both cases, all but one of the *P*. *vulgaris* accessions belongs to one of two major genetic groups (Fig 1D and 1E red vs. blue). The accessions in these two groups correspond precisely to the accessions in the two groups identified by PCA and cluster analysis. Once again, DUD_11 is the exception in that it appeared to contain genetic elements from both groups, consistent with it being a hybrid.

In accordance with the previous analyses, nucleotide diversity (π) was approximately four times greater between the red and blue clusters than within either cluster (Table 5). Although nucleotide diversity was significantly higher in the red cluster (P < 0.001, 1000 bootstrap replicates), the difference was not large.

**Table 5:** Nucleotide diversity within and between red and blue clusters.

	Mean π	π Standard Error	π 95% CI (bootstrap)
All 34 Accessions	0.00289	0.00006	(0.00275, 0.00303)
Between Red and Blue Clusters	0.00484	0.000074	(0.00481, 0.00487)
Within Red Cluster	0.00137	0.000027	(0.00132, 0.00142)
Within Blue Cluster	0.00110	0.000034	(0.00103, 0.00117)

### Relationship between morphological and genetic variation

We examined whether the two genetic clusters identified in the previous section are differentiated with respect to the morphological traits by performing a MANOVA and univariate ANOVAs in which the main effect was genetic cluster (the purported hybrid accession DUD_11 was excluded in from this analysis). In the multivariate analysis, the cluster effect was significant (Wilks Lambda = 0.3656, F = 2.54, df = 13, 19, P = 0.032).

The univariate analyses indicated that this differentiation was nominally significant for several traits: leaflet length, leaflet width, pod length, pod width, seed width, seed weight, and seeds per pod (Table 6, S4 Table, and S4 Fig). Each of these traits is larger in the blue cluster, suggesting that the blue cluster corresponds to large-seeded Andean accessions and the red cluster corresponds to small-seeded Mesoamerican accessions. On average, the between-cluster proportion of total variation for these traits is 0.393, indicating a not insubstantial divergence between clusters. For the remaining traits, the proportion of variation between clusters averages only 0.0328, with the proportion within clusters being 0.9672. When a sequential Bonferroni test was applied, leaflet length, pod width, seed width, and seed weight remained significant at an overall level of 0.05, with an average between-cluster proportion of variance equal to 0.483.

**Table 6:** Variance components (proportion of variance explained for the two different genetic clusters for 13 morphological traits).

Traits	Between-site Variance Component	Within site Variance Component	P
Days to flowering	0.015	0.985	.280
Plant height	0.000	1.000	.928
Leaflet length	0.458	0.542	.0015*
Leaflet width	0.309	0.691	.0136
Stem girth	0.000	1.000	.554
Pod length	0.229	0.771	.0347
Pod width	0.405	0.595	.0036*
Pod beak length	0.182	0.818	.057
Number seeds per pod	0.000	1.000	.686
Seed length	0.280	0.720	.0192
Seed width	0.433	0.567	.0023*
Hundred Seed weight	0.636	0.364	< .001*
Seed yield per plant	0.000	1.000	.450

P: P value for test of Cluster effect in ANOVA.

*Significant after sequential Bonferroni correction for 13 traits.

## Discussion

In Pakistan, the production of common bean has been centered on local landrace accessions cultivated by small-scale farmers mostly under low-input agriculture [22]. The traditional *P*. *vulgaris* landraces have been persistent in crop systems despite the import of seeds from other countries to meet local bean demand, presumably because of local cultural values and the inherent quality of local common bean genotypes [23]. The local accessions exhibit considerable morphological diversity, which reflects rich germplasm diversity owing to geographic and microclimatic variation in bean producing areas in the mountainous region of Kashmir [14]. In the current study, *P*. *vulgaris* accessions were characterized both morphologically and genetically to examine geographical patterns of variation.

Our survey of diversity among *P*. *vulgaris* bean cultivars in AJ&K, Pakistan has revealed two major patterns. The first is that there is little large-scale geographic differentiation, either genetically or in trait characteristics. Instead, most variation occurs within sampling sites. A similar lack of geographic differentiation has been reported in other countries [50, 51]. One likely explanation of this pattern is the tradition of sowing mixed seeds. Subsistence farmers tend to keep seed mixtures intact and impose little selection on their landraces, resulting in high local diversity [52]. In addition, variation within localities may be due to temporal heterogeneity associated with short-term climatic variation, with different varieties performing better in different seasons [53]. Finally, germplasm exchange among geographical regions may also contribute to this pattern [51, 54].

A second pattern is that most of the genetic variation in traits is between two major genetic clusters with similar geographic distributions, as has been found in previous studies in other countries [54–56]. Identical clustering was revealed by the STRUCTURE analysis, by cluster analysis and by Principal component analysis. The smaller cluster (Cluster A, blue) was comprised of 10 large-seeded accessions, based on mean HSW > 40g/100 seeds. These accessions also tended to have large leaves and pods. The 23 accessions in the larger cluster (Cluster B, red) had smaller seeds (< 30g/100 seeds). These two clusters presumably correspond to different geographic origin. Mesoamerican varieties tend to have small to medium sized seeds, while Andean varieties tend to have large seeds [57–59]. Although it would be useful to confirm this inference by comparing sequence similarity, it would thus seem that the large-seeded cultivars in our study are likely derived from the Andes, while those with smaller seeds are likely derived from Mesoamerica. Assuming that the relative proportions of large- and small-seeded cultivars in our study are representative of the proportions in AJ&K, Pakistan as a whole, there are more accessions belonging to Mesoamerican gene pool than Andean gene pool. The apparent preference for small-seeded accessions in the AJ&K region may be attributed to the desirable traits such as taste, seed size, growth habit, and seed colour. Such preferences have also been recorded in several other parts of the world including China [52, 60, 61], India [14, 62], Brazil [11] and Italy [63]. This distribution difference might be related to the germplasm introduction time, germplasm adaptation abilities, ecological types and consumer preferences.

Genetic diversity in the Mesoamerican samples is considerably less than that reported for land races from this region. Our estimate of π for Pakistani Mesoamerican derivatives (0.00137) is less than ½ to 1/7 that of Mesoamerican land races (0.0099, Mamidi et al. [64]; 0.0030, Bitocchi et al. [65]). Our estimate of π for Pakistani Andean derivatives (0.00110) is between estimates for Andean land races (0.0066, Mamidi et al. [64]; 0.00074, Bitocchi et al. [65]), but is only 1/6 of the higher estimate. While a small number of genes were used in these previous studies to estimate π in Andean accessions (13 and 5 for Mamidi et al. [64] and Bitocchi et al. [65], respectively) we believe the reduced diversity of Mesoamerican derivatives in Pakistan is likely real, and may also be true for Andean derivatives. This reduction suggests severe bottlenecks occurred during dispersal of the common bean to Pakistan. Additionally, it suggests genetic variation for crop improvement, especially of Mesoamerican derivates, in Pakistan may be severely limited and that breeders should consider introducing genetic material from other regions.

Two accessions in this study fall outside the two major genetic clusters. One is NGD-13, which is not surprising because this accession represented a different species i.e., *P*. *coccineus*. Of more interest is accession DUD_11 (S5 Table), which lies between the two main groups in the PCA analysis and in the dendrogram. The STRUCTURE analysis demonstrated that this accession consists of a mixture of genotypes characteristic of the two main clusters, suggesting that it represents a natural hybrid between one accession from each cluster. Unfortunately, because all trait values lie within the range of values for both clusters (S5 Table), traits of this accession are not informative about hybridization. This type of hybridization is not unexpected, given that accessions from both clusters frequently grow at the same locations. This type of hybridization has been found in *P*. *vulgaris* from other regions [66] as well as in CIAT core collections [67]. Such hybrid accessions may be of interest to breeders because they constitute germplasm bridging the Andean and Mesoamerican gene pools and thus may be helpful in improving Andean and Mesoamerican varieties [16].

Our study has revealed an interesting trend regarding growth form, as most of the accessions we examined were indeterminate climbers growing to the taller heights. This trait may be beneficial in Pakistan because beans tend to be grown as intercrops, often with maize, with the bean plants climbing the maize stalks. A tall stature may thus be an eco-physiological adaptation for attaining maximum light exposure and increasing their photosynthetic efficiency. The indeterminate climber growth habit is reported to be the best adapted for mountainous areas with cooler and wet climates [68] such as those found in the AJ&K region, Pakistan.

A comparative analysis of the literature reveals that our leaf size values are considerably higher than those reported by Bode et al. [69] and Loko et al. [70]. The difference in leaf area is significantly correlated with the geographical distribution of common bean genotypes as an adaptation in cooler agroclimatic zones. Consequently, cool temperature of the temperate mountainous environment facilitated the leaf expansion. Duncan and Hesketh [71] also reported greater leaf area in highland cold temperatures adapted maize genotypes as compared to lowland-adapted genotypes. It was also observed that large-seeded genotypes had large surface area. It has been reported that Andean lines exhibit larger leaves attributed to a selection pressure for larger leaves caused by cooler temperatures, which inversely affects the leaf thickness [72]. The lower leaf thickness also facilitates the tall climber genotypes to bear more leaves by reducing the leaf weight.

Our results also indicate relatively long Days to flowering in the investigated genotypes averaging as 65 days. Days to flowering is an important parameter which reflects the physiological response of plant growth mechanisms influenced by the climatic factors, including temperature and precipitation [70]. This trait may have evolved via changes in photoperiodism sensitivity and adaptation to the Cooler temperature of the Himalayan temperate region in which common bean landraces have been growing [72].

## Conclusion

The present study revealed considerable morphological and genetic variation among common bean accessions from Azad Jammu and Kashmir, Pakistan. There is little geographic differentiation either genetically or morphologically and much variation occurs locally within sites. Accessions were divided into two genetic groups that differed in seed length and width, hundred seed weight, and seed yeild, presumably reflecting Andean and Mesoamerican sources. One accession lies outside the two genetics groups and is more likely a hybrid between the two groups. The depauperate variation in Pakistani derivatives from Mesoamerica, and possibly from the Andes, suggests that breeding programs should include accessions from outside Pakistan.

## Supporting information

S1 Fig Histograms of heterozygosity and minor allele frequency for the variants we detected. (DOCX)

S2 Fig Correlations of means across years for the 13 morphological traits. Each point represents one accession.
(DOCX)

S3 Fig Evanno plot for determining the number of distinct genetic groups in the STRUCTURE analysis of 34 *P*. *vulgaris* accessions. (DOCX)

S4 Fig Box plots for morphological traits comparing accessions in the two genetic clusters. Colors of clusters correspond to colors of clusters in Fig 1.
(DOCX)

S1 Table Inventory of Phaseolus accessions and geographical characters of sampling sites in AJK. (DOCX)

S2 Table ANOVA for testing differences in morphological traits among accessions in years 2017 and 2018 for 34 Phaseolus vulgaris accessions. (DOCX)

S3 Table Mean and Standard error of morphological trait values. Means are averages across 2017 and 2018 for 34 Phaseolus vulgaris accessions.
(DOCX)

S4 Table Statistics for morphological traits by genetic cluster for 34 Phaseolus vulgaris accessions. A. Statistics for Blue cluster. B. Statistics for Red cluster.
(DOCX)

S5 Table Trait values for accession DUD-11 compared to ranges of values for Red (Mesoamerican) and Blue (Andean) clusters. Low: lowest value of trait among accessions within cluster. High: Highest value of trait among accession within clusters.
(DOCX)
