# Supplementary material for: Morphological and molecular characterization of variation in common bean (Phaseolus vulgaris L.) germplasm from Azad Jammu and Kashmir, Pakistan
Source: PLoS One. 2022 Apr 26;17(4):e0265817. doi: 10.1371/journal.pone.0265817 (PMC9041810; doi:10.1371/journal.pone.0265817)
Supplement: S2 Fig — Each point represents one accession. (DOCX) [file pone.0265817.s002.docx]

**
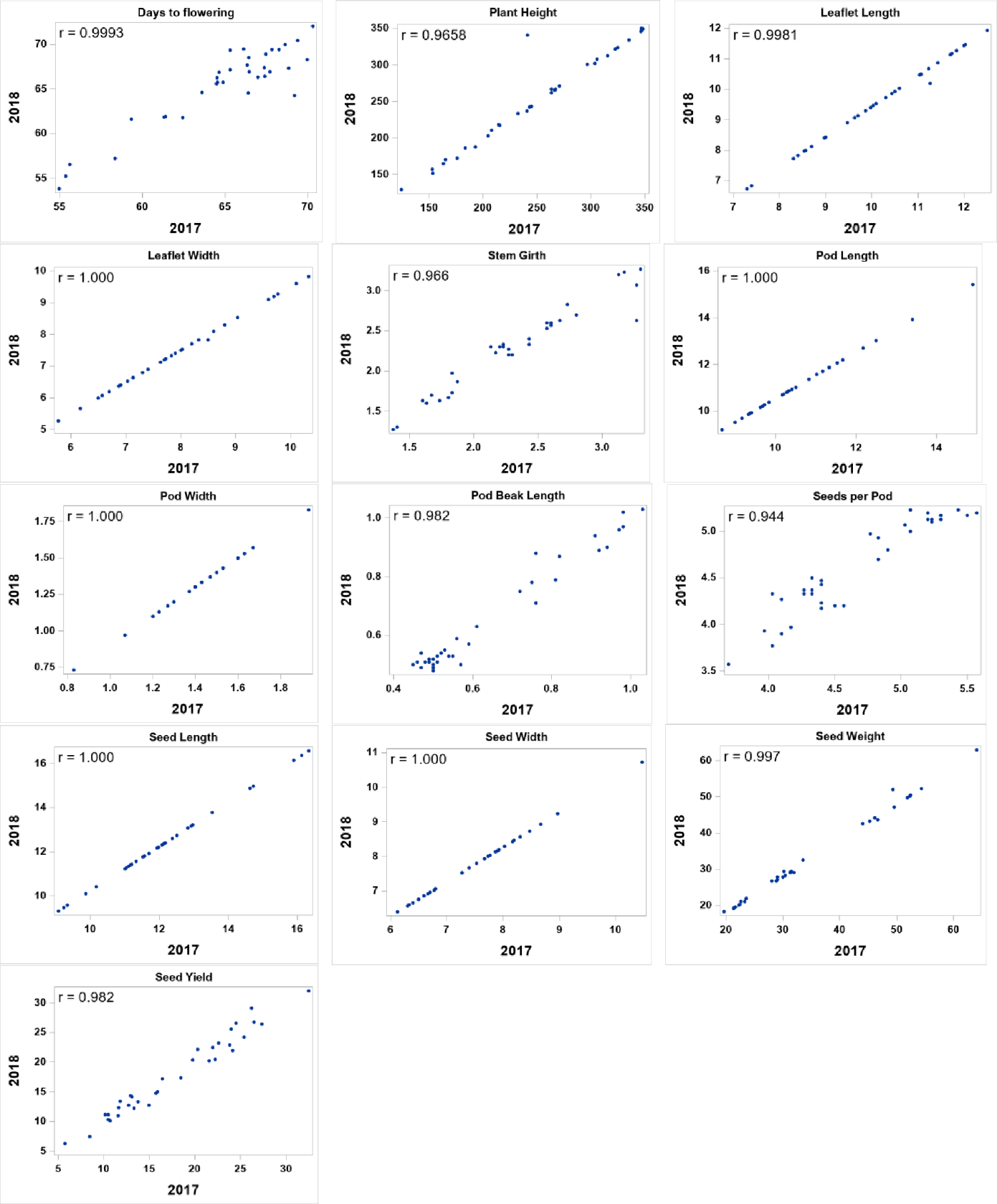
**

**S2 Fig.** Correlations of means across years for the 13 morphological traits. Each point represents one accession.
