# Supplementary material for: Morphological and molecular characterization of variation in common bean (Phaseolus vulgaris L.) germplasm from Azad Jammu and Kashmir, Pakistan
Source: PLoS One. 2022 Apr 26;17(4):e0265817. doi: 10.1371/journal.pone.0265817 (PMC9041810; doi:10.1371/journal.pone.0265817)
Supplement: S3 Fig — (DOCX) [file pone.0265817.s003.docx]

**S3 Fig.** Evanno plot for determining the number of distinct genetic groups in the STRUCTURE analysis of 1 *P. coccineus* and 34 *P. vulgaris* accessions.
