# Supplementary material for: Morphological and molecular characterization of variation in common bean (Phaseolus vulgaris L.) germplasm from Azad Jammu and Kashmir, Pakistan
Source: PLoS One. 2022 Apr 26;17(4):e0265817. doi: 10.1371/journal.pone.0265817 (PMC9041810; doi:10.1371/journal.pone.0265817)
Supplement: S4 Fig — Colors of clusters correspond to colors of clusters in Fig 1. (DOCX) [file pone.0265817.s004.docx]

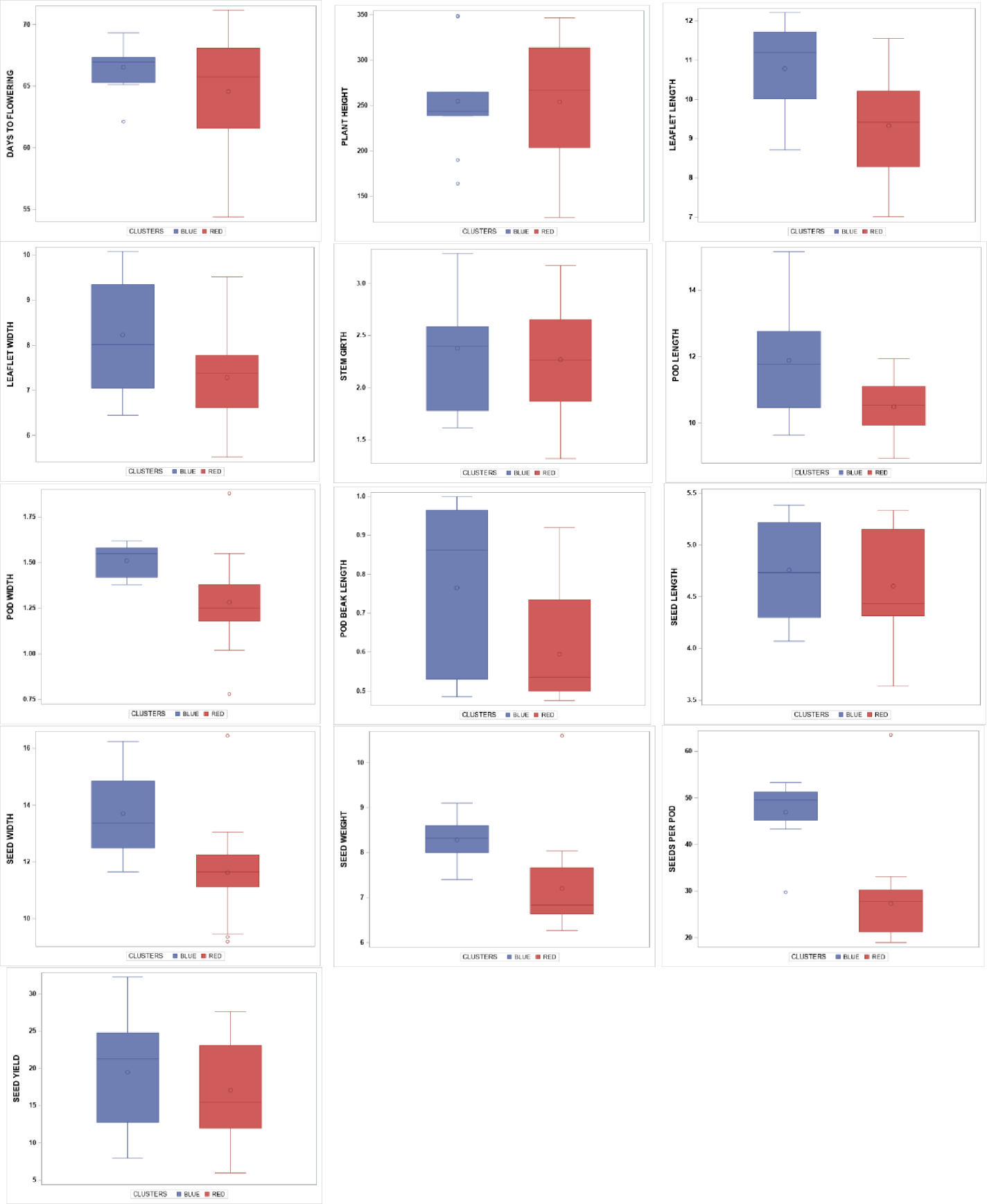


**S4 Fig.** Box plots for morphological traits comparing accessions in the two genetic clusters. Colors of clusters correspond to colors of clusters in Figure 1
