# Supplementary material for: Morphological and molecular characterization of variation in common bean (Phaseolus vulgaris L.) germplasm from Azad Jammu and Kashmir, Pakistan
Source: PLoS One. 2022 Apr 26;17(4):e0265817. doi: 10.1371/journal.pone.0265817 (PMC9041810; doi:10.1371/journal.pone.0265817)
Supplement: S1 Table — (DOCX) [file pone.0265817.s005.docx]

**S1 Table.** Inventory of *Phaseolus* accessions and geographical characters of sampling sites in AJK

| **AccNo.** | **Accession code** | **Species** | **Local name** | **Seed color** | **Seed Shape** | **District** | **Sampling site** | **Altitude (m)** | **Latitude (N)** | **Longitud (E)** |
| --- | --- | --- | --- | --- | --- | --- | --- | --- | --- | --- |
| 1 | NGD_13 | *P. coccineus* | Motty moth | Black+ pinkish | Kidney shape | Neelum | Nagder | 2244 | 34˚40'47" | 73˚56'14" |
| 2 | NGD_1 | *P. vulgaris* | Golay daby | Beige+ Maroon | Round | Neelum | Nagder | 2242 | 34˚40'47" | 73˚56'14" |
| 3 | DUD_1 | *P. vulgaris* | Chitty barhy moth | White | Kidney shape | Neelum | Dodnyal | 1812 | 34˚42'08" | 74˚06'25" |
| 4 | DUD_2 | *P. vulgaris* | Kaly moth | Black | Truncate festigate | Neelum | Dodnyal | 1807 | 34˚42'08" | 74˚06'25" |
| 5 | DUD_4 | *P. vulgaris* | Pelay moth | Yellow | Kidney shape | Neelum | Dodnyal | 1807 | 34˚42'08" | 74˚06'25" |
| 6 | DUD_8 | *P. vulgaris* | Changay | Maroon+ pale brown | Cylindrical | Neelum | Thunda pani | 2344 | 34˚40'30" | 74˚05'31" |
| 7 | DUD_11 | *P. vulgaris* | Golay | Light pink+ maroon | Cuboid | Neelum | Thunda pani | 2344 | 34˚40'30" | 74˚05'31" |
| 8 | DUD_12 | *P. vulgaris* | Ratty golay | Red | Cuboid | Neelum | Thunda pani | 2344 | 34˚40'30" | 74˚05'31" |
| 9 | KEL_2 | *P. vulgaris* | Golay | Maroon+ white | Oval | Neelum | Kel | 2152 | 34˚49'27" | 74˚21'07" |
| 10 | KEL_3 | *P. vulgaris* | - | Black | Kidney shape | Neelum | Kel | 2152 | 34˚49'27" | 74˚21'07" |
| 11 | KEL_5 | *P. vulgaris* | Chittay moth | White | Oval | Neelum | Kel | 2152 | 34˚49'27" | 74˚21'07" |
| 12 | KEL_8 | *P. vulgaris* | Chanjay | Cream+ maroon | Kidney shape | Neelum | Kel | 2152 | 34˚49'27" | 74˚21'07" |
| 13 | KEL_11 | *P. vulgaris* | Ratty mothy | Red | Truncate festigate | Neelum | Kel | 2152 | 34˚49'27" | 74˚21'07" |
| 14 | KEL_14 | *P. vulgaris* | - | Golden yellow | Cylindrical | Neelum | Arang Kel | 2379 | 34˚48'31" | 74˚21'06" |
| 15 | HAL_2 | *P. vulgaris* | - | Cream+ maroon | Oval | Neelum | Halmat | 2269 | 34˚45'09" | 74˚39'34" |
| 16 | HAL_6 | *P. vulgaris* | Shoray | Black | Truncate festigate | Neelum | Halmat | 2269 | 34˚45'09" | 74˚39'34" |
| 17 | LPA_1 | *P. vulgaris* | Chittay moth | White | Kidney shape | Hattian(Leepa valley) | Gai pora | 1832 | 34˚18'59" | 73˚51'01" |
| 18 | LPA_3 | *P. vulgaris* | Manjay | Pale brown | Cuboid | Hattian(Leepa valley) | Gai pora | 1832 | 34˚18'59" | 73˚51'01" |
| 19 | LPA_4 | *P. vulgaris* | Ratty moth | Dark red | Truncate festigate | Hattian(Leepa valley) | Gai pora | 1839 | 34˚18'41" | 73˚51'00" |
| 20 | LPA_5 | *P. vulgaris* | - | Black | Kidney shape | Hattian(Leepa valley) | Gai pora | 1839 | 34˚18'41" | 73˚51'00" |
| 21 | LPA_6 | *P. vulgaris* | Pelay moth | Yellow | Kidney shape | Hattian(Leepa valley) | Nokot | 2145 | 34˚18'23" | 73˚54'31" |
| 22 | LPA_9 | *P. vulgaris* | Dabi | Black | Kidney shape | Hattian(Leepa valley) | Nokot | 2145 | 34˚18'23" | 73˚54'31" |
| 23 | MAC_2 | *P. vulgaris* | - | Maroon | Kidney shape | Muzaffarabad | Machiyara | 2080 | 34˚29'58" | 73˚37'02" |
| 24 | MAC_3 | *P. vulgaris* | Golay daby | Beige+ maroon | Round | Muzaffarabad | Machiyara | 2080 | 34˚29'58" | 73˚37'02" |
| 25 | MAC_4 | *P. vulgaris* | Chittay moth | White | Kidney shape | Muzaffarabad | Machiyara | 2080 | 34˚29'58" | 73˚37'02" |
| 26 | FK_1 | *P. vulgaris* | Ratta lobia | Dark red | Kidney shape | Haveli (Farwad Kahuta) | Khurshidabad | 1705 | 33˚55'24" | 74˚10'26" |
| 27 | FK_2 | *P. vulgaris* | - | Beige+ olive | Kidney shape | Haveli (Farwad Kahuta) | Khurshidabad | 1705 | 33˚55'24" | 74˚10'26" |
| 28 | FK_3 | *P. vulgaris* | Dabi | Pale brown+ Black | Kidney shape | Haveli (Farwad Kahuta) | Khurshidabad | 1705 | 33˚55'24" | 74˚10'26" |
| 29 | FK_4 | *P. vulgaris* | - | Beige+ olive | Oval | Haveli (Farwad Kahuta) | Kirni | 1744 | 33˚56'27" | 74˚13'19" |
| 30 | FK_7 | *P. vulgaris* | - | Beige+ olive | Kidney shape | Haveli (Farwad Kahuta) | Kirni | 1744 | 33˚56'27" | 74˚13'19" |
| 31 | FK_8 | *P. vulgaris* | Manjay | Brown+ dark brown | Cuboid | Haveli (Farwad Kahuta) | Kirni | 1744 | 33˚56'27" | 74˚13'19" |
| 32 | FK_9 | *P. vulgaris* | Dabi | Purplish pink+ black | Kidney shape | Haveli (Farwad Kahuta) | Kirni | 1744 | 33˚56'27" | 74˚13'19" |
| 33 | RWK_1 | *P. vulgaris* | Ratta lobia | Dark red | Kidney shape | Poonch (Rawalakot) | Chhota gala | 1667 | 33˚49'21" | 73˚48'13" |
| 34 | RWK_2 | *P. vulgaris* | - | Cream+ maroon | Kidney shape | Poonch (Rawalakot) | Chhota gala | 1667 | 33˚49'21" | 73˚48'13" |
| 35 | BG_1 | *P. vulgaris* | Ratta lobia | Red | Kidney shape | Bagh | Saver | 1398 | 34˚01'31" | 73˚48'57" |
