# Supplementary material for: Morphological and molecular characterization of variation in common bean (Phaseolus vulgaris L.) germplasm from Azad Jammu and Kashmir, Pakistan
Source: PLoS One. 2022 Apr 26;17(4):e0265817. doi: 10.1371/journal.pone.0265817 (PMC9041810; doi:10.1371/journal.pone.0265817)
Supplement: S2 Table — (DOCX) [file pone.0265817.s006.docx]

**S2 Table.** ANOVA for testing differences in morphological traits among accessions in years 2017 and 2018 for 34 *Phaseolus vulgaris* accessions.

| **2017** | | | | | | | | | | | | | | | |
| --- | --- | --- | --- | --- | --- | --- | --- | --- | --- | --- | --- | --- | --- | --- | --- |
|  | | | | | | | | | | | | | | | |
| **Source of variation** | **Df** | | **PH** | **LL** | **LW** | **SG** | **PL** | **PW** | **PBL** | **SL** | **SW** | **HSW** | **SPP** | **SYPP** | **DTF** |
| Blocks | 2 | | 20.500 | 1.390 | 1.051 | 0.589 | 0.409 | 0.005 | 0.002 | 2.618 | 0.227 | 24.512 | 0.032 | 7.459 | 16.086 |
| Accessions | 33 | | 12588.100 | 5.933 | 4.232 | 0.867 | 5.162 | 0.133 | 0.111 | 9.722 | 2.680 | 449.492 | 0.798 | 131.153 | 51.594 |
| Error | 66 | | 9.000 | 0.809 | 0.924 | 0.043 | 0.683 | 0.019 | 0.005 | 0.480 | 0.181 | 6.032 | 0.093 | 0.786 | 1.221 |
|  |  | |  |  |  |  |  |  |  |  |  |  |  |  |  |
| **2018** | | | | | | | | | | | | | | | |
|  | | | | | | | | | | | | | | | |
| **Source of variation** | **Df** | **PH** | | **LL** | **LW** | **SG** | **PL** | **PW** | **PBL** | **SL** | **SW** | **HSW** | **SPP** | **SYPP** | **DTF** |
| Blocks | 2 | 23.300 | | 1.484 | 2.031 | 0.082 | 0.312 | 0.005 | 0.011 | 1.838 | 0.544 | 38.477 | 0.101 | 6.417 | 6.811 |
| Genotypes | 33 | 13152.600 | | 5.849 | 4.214 | 0.822 | 5.162 | 0.133 | 0.108 | 9.722 | 2.680 | 463.075 | 0.743 | 134.462 | 58.8259 |
| Error | 66 | 2.800 | | 0.838 | 0.935 | 0.036 | 0.683 | 0.019 | 0.006 | 0.480 | 0.181 | 5.843 | 0.093 | 2.319 | 2.2554 |

Key: PH=Plant height; LL= Leaflet length; LW= Leaflet width; SG = Stem girth; PL= Pod length (cm); PW= Pod width (cm); PBL= Pod beak length (cm); SL= Seed length (mm); SW= Seed width (mm); HSW=Hundred seed weight (g), SPP=Seed per pod, DTF=Day to flowering, SYPP= Seed yield per plant.
