# Supplementary material for: Morphological and molecular characterization of variation in common bean (Phaseolus vulgaris L.) germplasm from Azad Jammu and Kashmir, Pakistan
Source: PLoS One. 2022 Apr 26;17(4):e0265817. doi: 10.1371/journal.pone.0265817 (PMC9041810; doi:10.1371/journal.pone.0265817)
Supplement: S3 Table — Means are averages across 2017 and 2018 for 34 Phaseolus vulgaris accessions. (DOCX) [file pone.0265817.s007.docx]

**S3 Table.** Mean and SE of trait values. Means are averages across 2017 and 2018 for 34 *Phaseolus vulgaris* accessions.

| **District** | **Site** | **Accessions** | **DTF** | **PH** | **LL** | **LW** | **SG** | **PL** | **PW** | **PBL** | **SPP** | **SL** | **SW** | **HSW** | **SYPP** |
| --- | --- | --- | --- | --- | --- | --- | --- | --- | --- | --- | --- | --- | --- | --- | --- |
| Neelum | Dodnyal | DUD-1 | 68.83±0.63 | 346.86±0.14 | 11.41±0.16 | 9.45±0.12 | 3.17±0.02 | 9.66±0.140 | 1.88±0.005 | 0.92±0.001 | 3.35±0.008 | 16.45±0.02 | 10.60±0.034 | 63.52±0.605 | 11.27±0.19 |
| Neelum | Dodnyal | DUD-2 | 61.66±0.14 | 270.66±0.00 | 8.25±0.157 | 6.65±0.125 | 1.87±0.000 | 11.80±0.146 | 1.02±0.005 | 0.53±0.002 | 4.88±0.005 | 9.98±0.02 | 6.26±0.03 | 20.60±2.29 | 13.645±0.59 |
| Neelum | Dodnyal | DUD-4 | 61.58±0.16 | 346.13±0.080 | 10.15±0.157 | 7.78±0.125 | 2.75±0.005 | 10±0.146 | 1.35±0.005 | 0.73±0.000 | 5.33±0.05 | 12.08±0.02 | 7.86±0.03 | 30.5±3.92 | 23.35±0.378 |
| Neelum | Dodnyal | DUD-8 | 67.33±0.26 | 238.81±7.88 | 11.55±0.157 | 9.35±0.125 | 3.28±0.00 | 15.16±0.140 | 1.38±0.005 | 0.82±0.007 | 5.21±0.01 | 14.85±0.029 | 7.40±0.034 | 45.21±4.4 | 32.26±0.097 |
| Neelum | Dodnyal | DUD-11 | 66.23±1.74 | 184.76±2.71 | 11.15±0.157 | 7.45±0.125 | 2.27±0.000 | 9.63±0.140 | 1.48±0.005 | 1.03±0.000 | 4.18±0.045 | 12.05±0.026 | 8.16±0.03 | 44.20±2.00 | 13.64±1.066 |
| Neelum | Dodnyal | DUD-12 | 62.13±0.180 | 264.93±5.120 | 10.95±0.157 | 7.95±0.125 | 2.38±0.005 | 9.63±0.140 | 1.62±0.005 | 0.54±0.000 | 4.41±0.00 | 13.65±0.029 | 8.3±0.034 | 48.38±2.8 | 10.41±0.010 |
| Dodnyal Average | | | 64.63±1.315 | 275.36 ±25.68 | 10.57 ±0.507 | 8.10±0.44 | 2.62 ±0.22 | 10.98 ± 0.904 | 1.45± 0.118 | 0.76 ±0.082 | 4.61±0.26 | 13.18±0.936 | 8.09 ±0.58 | 42.08±6.0 | 17.43±3.516 |
| Neelum | Kel | KEL-2 | 65.3±0.370 | 244.03±2.000 | 10.31±0.16 | 7.95±0.125 | 1.78±0.005 | 11.3±0.146 | 1.58±0.005 | 0.50±0.002 | 4.28±0.02 | 11.65±0.029 | 9.1±0.034 | 45.18±2.0 | 15.21±0.451 |
| Neelum | Kel | KEL-3 | 55.32±0.005 | 167.76±12.1 | 8.28±0.162 | 6.25±0.125 | 2.25±0.005 | 10.6±0.146 | 1.15±0.005 | 0.57±0.000 | 5.15±0.013 | 11.45±0.029 | 6.63±0.03 | 20.26±2.2 | 11.96±0.266 |
| Neelum | Kel | KEL-5 | 68.08±1.125 | 126.78±12.30 | 8.28±0.162 | 5.92±0.125 | 2.95±0.140 | 8.93±0.205 | 0.78±0.005 | 0.49±0.000 | 4.35±0.045 | 9.18±0.026 | 6.46±0.03 | 18.97±0.98 | 5.97±0.151 |
| Neelum | Kel | KEL-8 | 67±0.898 | 243.86±1.17 | 9.18±0.162 | 6.78±0.125 | 2.23±0.00 | 13.66±0.140 | 1.42±0.005 | 0.96±0.000 | 5.2±0.000 | 16.01±0.02 | 8±0.034 | 51.45±1.80 | 21.31±1.566 |
| Neelum | Kel | KEL11 | 65.03±0.63 | 216.55±1.805 | 9.58±0.162 | 6.88±0.125 | 1.61±0.00 | 9.43±0.140 | 1.38±0.005 | 0.76±0.000 | 3.9±0.034 | 11.28±0.02 | 7.4±0.034 | 28.11±1.5 | 20.09±0.205 |
| Neelum | Kel | KEL14 | 68.2±1.066 | 190.09±13.8 | 10.01±0.16 | 7.05±0.125 | 2.41±0.00 | 12.43±0.140 | 1.42±0.005 | 0.90±0.000 | 4.33±0.000 | 14.75±0.029 | 7.8±0.034 | 43.33±0.98 | 7.94±0.541 |
| Kel Average | | | 64.82 ±1.978 | 198.18±18.81 | 9.27±0.351 | 6.80±0.28 | 2.20±0.19 | 11.06± 0.733 | 1.28± 0.116 | 0.70 ±0.084 | 4.53±0.21 | 12.38±1.027 | 7.56±0.39 | 34.55 ±5.66 | 13.75±2.562 |
| Neelum | Halmat | HAL-2 | 66.72±0.125 | 242.73±0.274 | 11.71±0.16 | 8.08±0.125 | 1.61±0.00 | 11.93±0.140 | 1.55±0.005 | 0.97±0.000 | 5.33±0.020 | 12.61±0.02 | 8.6±0.034 | 51.23±2.2 | 22.18±0.140 |
| Neelum | Halmat | HAL-6 | 56.1±0.442 | 154.95±6.845 | 7.01±0.162 | 5.52±0.125 | 1.35±0.005 | 11.93±0.140 | 1.15±  0.005 | 0.73±0.001 | 4.76±0.00 | 11.31±0.02 | 6.73±0.03 | 20.33±2.26 | 13.84±2.464 |
| Halmat Average | | | 61.41±5.310 | 198.84±43.89 | 9.36 ±2.350 | 6.80±1.28 | 1.48 ±0.13 | 11.93± 0.000 | 1.35 ±0.200 | 0.85± 0.120 | 5.04±0.28 | 11.96±0.650 | 7.66±0.93 | 35.78±15.4 | 18.01±4.17 |
| Neelum | Nagder | NGD-1 | 65.13±0.720 | 48.83±0.720 | 12.21±0.16 | 10.08±0.12 | 2.55±0.001 | 10.46±0.140 | 1.55±0.005 | 1±0.001 | 5.05±0.001 | 12.48±0.02 | 8.43±0.03 | 50.66±3.56 | 24.8±0.720 |
| Nagder Average | | | 65.13±0.720 | 348.83±0.720 | 12.21±0.16 | 10.08±0.12 | 2.55±0.001 | 10.46±0.140 | 1.55±0.005 | 1±0.001 | 5.05±0.001 | 12.48±0.02 | 8.43±0.03 | 50.66±3.56 | 24.8±0.720 |
| Neelum Average | | | 63.99 ±0.869 | 255.30±36.05 | 10.35 ±0.686 | 7.94 ±0.77 | 2.21±0.26 | 11.11±0.305 | 1.41± 0.058 | 0.83± 0.065 | 4.81±0.13 | 12.50±0.253 | 7.94 ±0.20 | 40.77±3.68 | 18.49±2.303 |
| Muzaffarabad | Machiyara | MAC-2 | 68.63±1.280 | 321.79±0.43 | 8.41±0.162 | 6.32±0.125 | 2.28±0.005 | 10.53±0.140 | 1.48±0.000 | 0.49±0.000 | 4.31±0.01 | 12.18±0.02 | 7.4±0.034 | 30.43±2.26 | 10.83±0.245 |
| Muzaffarabad | Machiyara | MAC-3 | 69.31±0.93 | 348.33±5.314 | 11.45±0.157 | 9.85±0.125 | 2.58±0.00 | 11.6±0.146 | 1.55±0.005 | 0.48±0.001 | 4.3±0.002 | 13.08±0.02 | 8.8±0.034 | 50.8±2.290 | 21.21±1.748 |
| Muzaffarabad | Machiyara | MAC-4 | 65.75±2.509 | 265.86±0.37 | 9.75±0.157 | 7.15±0.125 | 2.26±0.00 | 10.66±0.140 | 1.25±0.005 | 0.50±0.000 | 3.95±0.001 | 11.81±0.02 | 6.73±0.03 | 27.8±2.290 | 10.65±0.510 |
| Machiyara Average | | | 67.89 ±1.092 | 311.99±24.30 | 9.87±0.878 | 7.77±1.06 | 2.37 ±0.10 | 10.93±0.335 | 1.42± 0.091 | 0.49± 0.006 | 4.18 ±0.11 | 12.36±0.377 | 7.64 ±0.60 | 36.34±7.26 | 14.23±3.491 |
| Muzaffarabad Average | | | 67.89 ±1.092 | 311.99±24.30 | 9.87±0.878 | 7.77±1.06 | 2.37 ±0.10 | 10.93±0.335 | 1.42± 0.091 | 0.49± 0.006 | 4.18 ±0.11 | 12.36±0.377 | 7.64 ±0.60 | 36.34±7.26 | 14.23±3.491 |
| Poonch | Chhota Gala | RWK-1 | 69.93±0.57 | 298.63±10.58 | 8.11±0.162 | 6.62±0.125 | 2.65±0.001 | 9.6±0.146 | 1.55±0.005 | 0.84±0.001 | 4.38±0.06 | 13.05±0.029 | 8±0.034 | 27.43±0.80 | 13.55±0.088 |
| Poonch | Chhota Gala | RWK-2 | 67.33±8.28 | 262.38±0.845 | 11.75±0.157 | 8.78±0.125 | 3.2±0.002 | 12.76±0.140 | 1.58±0.005 | 0.92±0.000 | 5.38±0.06 | 16.25±0.029 | 8.33±0.03 | 53.3±2.290 | 26.6±0.029 |
| Chhota Gala Average | | | 68.63±1.300 | 280.50±18.12 | 9.93± 1.818 | 7.70±1.08 | 2.92±0.27 | 11.18± 1.583 | 1.56± 0.015 | 0.88± 0.040 | 4.88±0.50 | 14.65±1.600 | 8.16± 0.168 | 40.36±12.9 | 20.07±6.525 |
| Poonch Average | | | 68.63±  1.300 | 280.50±18.12 | 9.93± 1.818 | 7.70± 1.080 | 2.92±0.27 | 11.18± 1.583 | 1.56± 0.015 | 0.88± 0.040 | 4.88±0.50 | 14.65±1.600 | 8.16±0.16 | 40.36 ±12.9 | 20.07±6.525 |
| Hattian | Gaipora | LPA-1 | 67.37±0.000 | 174.41±5.88 | 9.68±0.162 | 7.38±0.125 | 2.21±0.01 | 10.66±0.140 | 1.42±0.005 | 0.5±0.000 | 4±0.020 | 12.28±0.02 | 6.9±0.034 | 28.4±0.720 | 12.73±0.000 |
| Hattian | Gaipora | LPA-3 | 67.81±5.67 | 216.28±7.069 | 9.81±0.162 | 7.48±0.125 | 1.32±0.005 | 9.93±0.140 | 1.22±0.005 | 0.51±0.000 | 4.85±0.005 | 10.28±0.02 | 6.73±0.03 | 21.85±1.12 | 16.8±0.289 |
| Hattian | Gaipora | LPA-4 | 65.38±1.56 | 334.53±0.650 | 10.75±0.157 | 7.75±0.125 | 1.68±0.005 | 11.6±0.146 | 1.48±0.005 | 0.8±0.000 | 4.87±0.020 | 12.95±0.029 | 8.03±0.03 | 30.23±2.26 | 15.44±0.336 |
| Hattian | Gaipora | LPA-5 | 65.48±1.67 | 203.71±2.06 | 9.41±0.162 | 7.58±0.125 | 2.2±0.002 | 10.76±0.140 | 1.18±0.005 | 0.52±0.000 | 4.18±0.01 | 11.65±0.029 | 6.63±0.03 | 21.36±2.26 | 17.90±0.530 |
| Gaipora Average | | | 66.51 ±0.630 | 232.23±35.20 | 9.91±0.290 | 7.54 ±0.07 | 1.85±0.21 | 10.74±0.341 | 1.32± 0.074 | 0.58± 0.073 | 4.47±0.22 | 11.79±0.568 | 7.07±0.32 | 25.46±2.25 | 15.71±1.117 |
| Hattian | Nokot | LPA-6 | 64.1±0.500 | 291.06±3.22 | 8.01±0.162 | 6.65±0.125 | 1.9±0.010 | 11.43±0.140 | 1.35±0.005 | 0.48±0.000 | 5.16±0.00 | 12.05±2.290 | 7.9±0.034 | 30.3±0.029 | 23.06±2.376 |
| Hattian | Nokot | LPA-9 | 54.4±0.650 | 209.14±3.83 | 10.78±0.16 | 8.55±0.125 | 1.68±0.00 | 11.1±0.146 | 1.22±0.005 | 0.51±0.000 | 5.23±0.00 | 9.35±0.029 | 6.93±0.03 | 21.13±2.26 | 20.89±0.884 |
| Nokot Average | | | 59.25 ±4.850 | 250.10±40.96 | 9.40 ±1.385 | 7.60±0.95 | 1.79±0.10 | 11.26± 0.168 | 1.28± 0.065 | 0.49± 0.015 | 5.20±0.03 | 10.70±1.350 | 7.41±0.48 | 25.71±4.58 | 21.97±1.083 |
| Hattian Average | | | 62.88±3.632 | 241.17± 8.935 | 9.65±±0.258 | 7.57±0.02 | 1.82±0.03 | 11.00± 0.263 | 1.30± 0.020 | 0.53± 0.044 | 4.83±0.362 | 11.24±0.546 | 7.24±0.17 | 25.59±0.12 | 18.84±3.129 |
| Haveli | Khurshidabad | FK-1 | 71.16±1.49 | 266.91±0.16 | 8.68±0.162 | 6.88±0.125 | 2.25±0.005 | 10.1±0.146 | 1.32±0.005 | 0.47±0.001 | 4.32±0.005 | 12.25±0.029 | 7.66±0.03 | 30.23±2.26 | 26.9±0.405 |
| Haveli | Khurshidabad | FK-2 | 66.73±12.15 | 324.19±0.378 | 11.55±0.157 | 9.52±0.125 | 2.58±0.00 | 10.56±0.140 | 1.35±0.005 | 0.58±0.000 | 4.35±0.001 | 12.61±0.02 | 7.53±0.03 | 33.1±0.442 | 10.41±0.135 |
| Haveli | Khurshidabad | FK-3 | 57.8±0.650 | 313.98±2.645 | 9.18±0.162 | 7.48±0.125 | 2.78±0.005 | 10.1±0.146 | 1.18±0.005 | 0.53±0.000 | 5.18±0.005 | 11.11±0.02 | 6.63±0.03 | 21.23±2.26 | 24.74±1.296 |
| Khurshidabad Average | | | 65.23 ±3.931 | 301.69 ±17.63 | 9.80 ±0.884 | 7.96±0.79 | 2.53±0.15 | 10.25± 0.155 | 1.28± 0.052 | 0.53± 0.030 | 4.61±0.28 | 11.99±0.452 | 7.27±0.324 | 28.19±3.57 | 20.68±5.175 |
| Haveli | Kirni | FK-4 | 69.13±1.394 | 302.75±1.445 | 9.35±0.157 | 7.65±0.125 | 2.6±0.000 | 10.43±0.140 | 1.18±0.005 | 0.49±0.000 | 4.43±0.00 | 9.45±0.029 | 6.43±0.03 | 28.9±2.290 | 22.9±0.168 |
| Haveli | Kirni | FK-7 | 66.65±0.205 | 232.81±0.684 | 10.73±0.572 | 8.16±0.22 | 2.38±0.005 | 11.6±0.146 | 1.35±0.005 | 0.62±0.000 | 5.03±0.00 | 11.21±0.02 | 6.83±0.036 | 29.4±2.290 | 27.63±4.118 |
| Haveli | Kirni | FK-8 | 67.46±2.142 | 152.61±2.06 | 6.83±0.162 | 5.52±0.125 | 2.28±0.005 | 10.43±0.140 | 1.02±0.005 | 0.5±0.000 | 4.41±0.014 | 11.15±0.029 | 6.53±0.036 | 22.75±1.44 | 12.57±1.280 |
| Haveli | Kirni | FK-9 | 60.48±2.645 | 306.41±2.06 | 10.21±0.162 | 8.35±0.125 | 3.16±0.00 | 9.26±0.140 | 1.25±0.005 | 0.54±0.000 | 5.16±0.00 | 12.21±0.026 | 6.8±0.034 | 22.2±2.290 | 25.53±2.122 |
| Kirni Average | | | 65.93 ±1.890 | 248.64 ±36.21 | 9.35± 0.799 | 7.42±0.65 | 2.60 ±0.19 | 10.43±0.477 | 1.20± 0.069 | 0.53± 0.030 | 4.76±0.19 | 11.00±0.574 | 6.65±0.09 | 25.81±1.93 | 22.15±3.340 |
| Haveli Average | | | 65.58±0.350 | 275.17±26.52 | 9.58 ±0.226 | 7.69±0.269 | 2.57±0.03 | 10.34±0.089 | 1.24± 0.042 | 0.53± 0.004 | 4.69 ±0.07 | 11.50±0.493 | 6.96±0.31 | 27.00±1.18 | 21.42±0.737 |
| Bagh | Saver | BG-1 | 66.91±0.470 | 164.18±1.549 | 8.71±0.162 | 6.45±0.125 | 1.73±0.00 | 9.9±0.146 | 1.45±0.005 | 0.53±0.000 | 4.07±0.020 | 11.68±0.02 | 8.06±0.03 | 29.77±0.32 | 12.76±0.638 |
| Saver Average | | | 66.91±0.470 | 164.18±1.549 | 8.71±0.162 | 6.45±0.125 | 1.73±0.00 | 9.9±0.146 | 1.45±0.005 | 0.53±0.000 | 4.07±0.020 | 11.68±0.02 | 8.06±0.03 | 29.77±0.32 | 12.76±0.638 |
| Bagh Average | | | 66.91±0.470 | 164.18±1.549 | 8.71±0.162 | 6.45±0.125 | 1.73±0.00 | 9.9±0.146 | 1.45±0.005 | 0.53±0.000 | 4.07±0.020 | 11.68±0.02 | 8.06±0.03 | 29.77±0.32 | 12.76±0.638 |

Key: PH=Plant height; LL= Leaflet length; LW= Leaflet width; SG = Stem girth; PL= Pod length (cm); PW= Pod width (cm); PBL= Pod beak length (cm); SL= Seed length (mm); SW= Seed width (mm); HSW=Hundred seed weight (g), SPP=Seed per pod, DTF=Day to flowering, SYPP= Seed yield per plant.
