# Supplementary material for: Morphological and molecular characterization of variation in common bean (Phaseolus vulgaris L.) germplasm from Azad Jammu and Kashmir, Pakistan
Source: PLoS One. 2022 Apr 26;17(4):e0265817. doi: 10.1371/journal.pone.0265817 (PMC9041810; doi:10.1371/journal.pone.0265817)
Supplement: S4 Table — A. Statistics for Blue cluster. B. Statistics for Red cluster. (DOCX) [file pone.0265817.s008.docx]

**S4 Table.** Statistics for morphological traits by genetic cluster for 34 *Phaseolus vulgaris* accessions. A. Statistics for Blue cluster. B. Statistics for Red cluster.

**A.**

| **Trait** | **N** | **Mean** | **Std Dev.** | **Minimum** | **Maximum** |
| --- | --- | --- | --- | --- | --- |
| \| Day to flowering \| \| --- \| \| Plant height \| \| Leaflet length \| \| Leaflet width \| \| Stem girth \| \| Pod length \| \| Pod width \| \| Pod beak length \| \| Seed length \| \| Seed width \| \| Hundred seed weight \| \| Seed per pod \| \| Seed yield per plant \| | \| 10 \| \| --- \| \| 10 \| \| 10 \| \| 10 \| \| 10 \| \| 10 \| \| 10 \| \| 10 \| \| 10 \| \| 10 \| \| 10 \| \| 10 \| \| 10 \| | \| 66.5380000 \| \| --- \| \| 254.8185000 \| \| 10.7860000 \| \| 8.2320000 \| \| 2.3780000 \| \| 11.8865000 \| \| 1.5100000 \| \| 0.7650000 \| \| 13.7035000  8.2835000 \| \| 46.9335000 \| \| 4.7580000 \| \|  \| \| 19.4705000 \| | \| 1.9742413 \| \| --- \| \| 58.4855779 \| \| 1.1810725 \| \| 1.2669456 \| \| 0.5721121 \| \| 1.7142784 \| \| 0.0839312 \| \| 0.2209575 \| \| 1.6860046  0.4946326 \| \| 6.8637227 \| \| 0.5182728 \| \|  \| \| 7.7081250 \| | \| 62.1300000 \| \| --- \| \| 164.1800000 \| \| 8.7150000 \| \| 6.4500000 \| \| 1.6150000 \| \| 9.6350000 \| \| 1.3800000 \| \| 0.4850000 \| \| 11.6500000  7.4000000 \| \| 29.7700000 \| \| 4.0700000 \| \|  \| \| 7.9400000 \| | \| 69.3150000 \| \| --- \| \| 348.8300000 \| \| 12.2150000 \| \| 10.0800000 \| \| 3.2850000 \| \| 15.1650000 \| \| 1.6200000 \| \| 1.0000000 \| \| 16.2500000  9.1000000 \| \| 53.3000000 \| \| 5.3850000 \| \|  \| \| 32.2600000 \| |

**B.**

| **Trait** |  | **N Mean** | **Std Dev.** | | **Minimum** | | **Maximum** |
| --- | --- | --- | --- | --- | --- | --- | --- |
| \| Day to flowering \| \| --- \| \| Plant height \| \| Leaflet length \| \| Leaflet width \| \| Stem girth \| \| Pod length \| \| Pod width \| \| Pod beak length \| \| Seed length \| \| Seed width \| \| Hundred seed weight \| \| Seed per pod \| \| Seed yield per plant \| | | \| 23 \| \| --- \| \| 23 \| \| 23 \| \| 23 \| \| 23 \| \| 23 \| \| 23 \| \| 23 \| \| 23 \| \| 23 \| \| 23 \| \| 23 \| \| 23 \| | \| 64.5617391 \| \| --- \| \| 254.1230435 \| \| 9.3289130 \| \| 7.2823913 \| \| 2.2691304 \| \| 10.4856522 \| \| 1.2830435 \| \| 0.5941304 \| \| 11.6254348 \| \| 7.2034783 \| \| 27.3543478 \| \| 4.6030435 \| \| 17.0736957 \| | \| 4.8610092 \| \| --- \| \| 68.6400942 \| \| 1.2710875 \| \| 1.0797473 \| \| 0.5266805 \| \| 0.8271642 \| \| 0.2154791 \| \| 0.1339238 \| \| 1.5256153 \| \| 0.9229233 \| \| 9.0454271 \| \| 0.4963085 \| \| 6.2382920 \| | | \| 54.4000000 \| \| --- \| \| 126.7800000 \| \| 7.0150000 \| \| 5.5200000 \| \| 1.3200000 \| \| 8.9350000 \| \| 0.7800000 \| \| 0.4750000 \| \| 9.1850000 \| \| 6.2650000 \| \| 18.9700000 \| \| 3.6350000 \| \| 5.9750000 \| | \| 71.1650000 \| \| --- \| \| 346.8650000 \| \| 11.5500000 \| \| 9.5200000 \| \| 3.1700000 \| \| 11.9350000 \| \| 1.8800000 \| \| 0.9200000 \| \| 16.4500000 \| \| 10.6000000 \| \| 63.5200000 \| \| 5.3350000 \| \| 27.6350000 \| |
