# Supplementary material for: Morphological and molecular characterization of variation in common bean (Phaseolus vulgaris L.) germplasm from Azad Jammu and Kashmir, Pakistan
Source: PLoS One. 2022 Apr 26;17(4):e0265817. doi: 10.1371/journal.pone.0265817 (PMC9041810; doi:10.1371/journal.pone.0265817)
Supplement: S5 Table — Low: lowest value of trait among accessions within cluster. High: Highest value of trait among accession within clusters. (DOCX) [file pone.0265817.s009.docx]

**S5 Table.** Trait values for accession DUD-11 compared to ranges of values for Red (Mesoamerican) and Blue (Andean) clusters. Low: lowest value of trait among accessions within cluster. High: Highest value of trait among accession within clusters.

|  | **Blue Cluster** | | **DUD-11** | **Red Cluster** | |
| --- | --- | --- | --- | --- | --- |
| **Trait** | **Low** | **High** |  | **Low** | **High** |
| Day to flowering | 62.13 | 69.315 | 66.23 | 54.4 | 71.165 |
| Plant height (cm) | 164.18 | 348.83 | 184.76 | 126.78 | 346.865 |
| Leaflet length (cm) | 8.715 | 12.215 | 11.15 | 7.015 | 11.55 |
| Leaflet width (cm) | 6.45 | 10.08 | 7.45 | 5.52 | 9.52 |
| Stem girth (cm) | 1.615 | 3.285 | 2.27 | 1.32 | 3.17 |
| Pod length (cm) | 9.635 | 15.165 | 9.63 | 8.935 | 11.935 |
| Pod width (cm) | 1.38 | 1.62 | 1.48 | 0.78 | 1.88 |
| Pod beak length | 0.485 | 1 | 1.03 | 0.475 | 0.92 |
| Seed length (mm) | 11.65 | 16.25 | 12.08 | 9.185 | 16.45 |
| Seed width (mm) | 7.4 | 9.1 | 8.16 | 6.265 | 10.6 |
| Hundred seed weight (g) | 29.77 | 53.3 | 44.23 | 18.97 | 63.52 |
| Seeds per pod | 4.07 | 5.385 | 4.18 | 3.635 | 5.335 |
| Seed yield per plant (g) | 7.94 | 32.26 | 13.64 | 5.975 | 27.635 |
